# Reporting guidelines for oncology research: helping to maximise the impact of your research

**DOI:** 10.1038/bjc.2017.407

**Published:** 2018-02-22

**Authors:** Angela MacCarthy, Shona Kirtley, Jennifer A de Beyer, Douglas G Altman, Iveta Simera

**Affiliations:** 1 UK EQUATOR Centre, Centre for Statistics in Medicine, Nuffield Department of Orthopaedics, Rheumatology and Musculoskeletal Sciences, University of Oxford, Botnar Research Centre, Windmill Road, Headington, Oxford OX3 7LD, UK

**Keywords:** reporting guidelines, oncology, EQUATOR Network, research waste

## Abstract

Many reports of health research omit important information needed to assess their methodological robustness and clinical relevance. Without clear and complete reporting, it is not possible to identify flaws or biases, reproduce successful interventions, or use the findings in systematic reviews or meta-analyses. The EQUATOR Network (http://www.equator-network.org/) promotes responsible reporting and the use of reporting guidelines to improve the accuracy, completeness, and transparency of health research. EQUATOR supports researchers by providing online resources and training. EQUATOR Oncology, a project funded by Cancer Research UK, aims to support cancer researchers reporting their research through the provision of online resources. In this article, our objective is to highlight reporting issues related to oncology research publications and to introduce reporting guidelines that are designed to aid high-quality reporting. We describe generic reporting guidelines for the main study types, and explain how these guidelines should and should not be used. We also describe 37 oncology-specific reporting guidelines, covering different clinical areas (e.g., haematology or urology) and sections of the report (e.g., methods or study characteristics); most of these are little-used. We also provide some background information on EQUATOR Oncology, which focuses on addressing the reporting needs of the oncology research community.

Health research studies must be reported clearly, accurately, and completely if they are to meaningfully enhance medical knowledge and inform clinical practice. Ambiguous, missing, or misleading information obscures how research was carried out and what was found, limiting critical assessment and impeding further use of published findings. It also wastes the financial and human resources invested in the research (Glasziou *et al*, 2014).

Readers cannot judge the robustness of the methodology used or the reliability of the findings if study methods are reported badly. Further, other researchers may be prevented from repeating the study (Goodman *et al*, 2016). Poor reporting of clinical details, inconsistent use of terminology and definitions, insufficient information about interventions, incomplete details of statistical methods, and inconsistent or missing reporting of adverse effects also hamper comparisons of findings across studies, which are necessary to determine the best options for patient care and disease prevention.

Oncology research suffers from the reporting inadequacies that afflict all health research. Table 1 gives some examples of the consequences of poor reporting in different study types in oncology.

Despite early calls by the World Health Organization (Unknown, 1979) and others (Nahum, 1979; Miller *et al*, 1981) to standardise how the results of cancer treatment studies are reported, many recent studies evaluating the quality of oncology clinical trial reporting have found biased and inconsistent reporting to be very common (Duff *et al*, 2010; Peron *et al*, 2012; Vera-Badillo *et al*, 2016). Basing decisions about patient care on incomplete and misleading research findings may have a profoundly negative impact on patients’ health and wellbeing.

These reporting problems are avoidable. In this paper, we introduce reporting guidelines – simple but effective tools supporting complete and transparent reporting – and highlight guidelines that are specifically useful for oncology research. We introduce the EQUATOR (Enhancing the QUAlity and Transparency Of health Research) Network programme, its online resources, and a new project, EQUATOR Oncology, which is collating oncology-specific resources.

## The EQUATOR network

The EQUATOR Network was set up in 2006 to support the implementation of reporting guidelines. Along with its database of reporting guidelines, the EQUATOR programme provides resources and toolkits to help researchers write complete and transparent health research papers. Resources are also provided for journal editors and peer reviewers to help ensure that published research is ‘fit for purpose’, that is, that it provides all of the information needed for its assessment and further use. The Network also organises events and conferences to raise awareness of poor reporting and its consequences, and offers training for researchers and editors to maximise the value of their published research. Ongoing work is expanding the scope of EQUATOR’s resources to also cover guidance for research planning. More information about the EQUATOR Network is available on our website (http://www.equator-network.org/about-us/).

EQUATOR’s scope is primarily studies of humans and pre-clinical animal research. We do not address laboratory research. Related resources for such research tend to target data sharing, such as MIAME for microarray experiments (Brazma *et al*, 2001; https://fairsharing.org/).

## Reporting guidelines

Reporting guidelines provide one solution to the widespread problems in research reporting. They are simple, efficient tools, most often in the form of a checklist, that help researchers to prepare manuscripts that contain all of the information required by readers and those that will use the research report.

Table 2 lists the generic guidelines for the main research study types. Most of these guidelines provide a reporting framework for a whole research paper and list the minimum information that authors should include within the paper so that their study can be fully understood, replicated if desired, and used to inform future research. Some of these guidelines also have extensions, offering additional guidance. The guidelines can also be used by peer reviewers to check that research reports are complete, accurate reflections of the research undertaken (Levine and Kressel, 2016).

The best known guidelines are the CONSORT Statement (checklist shown in Supplementary File 1) for reporting randomised controlled trials (Schulz *et al*, 2010) and the STROBE Statement (checklist shown in Supplementary File 2) for reporting observational studies (von Elm *et al*, 2007). Although reporting guidelines should not be used to critically appraise reports of research studies, they prompt authors to report the information needed for a complete critical appraisal.

Generic guidelines exist for reporting most major types of clinical and pre-clinical research. They provide an excellent starting point when writing up any study, including oncology research. Other reporting guidelines that provide guidance on reporting specific aspects of study methods, procedures, or medical conditions, including oncology-specific guidelines, can be found in the database of reporting guidelines on the EQUATOR Network website. The Network systematically collects and classifies all reporting guidelines to help researchers easily find the guidelines relevant to their work.

There is increasing interest in the publication of protocols for research studies. Guidelines are available for preparing protocols for randomised trials (Chan *et al*, 2013) and systematic reviews (Moher *et al*, 2015).

## EQUATOR Oncology

The UK EQUATOR Centre was awarded funding from Cancer Research UK to develop oncology-specific resources and activities to enhance the quality and transparency of published oncology research (http://www.equator-network.org/library/equator-oncology/).

We are reviewing the literature on the quality of reporting of clinical trials and observational studies in oncology. In the course of our work, we are collating published literature on oncology research methodology and reporting, which we regularly make available through the EQUATOR Oncology Current Awareness Bulletin.

The project focuses on research reporting, but in the next phase will be expanded to include guidance for efficient research planning and design. Ensuring robustness in the planning of any research project is the first condition for obtaining reliable research findings. For example, writing a detailed protocol documenting the study design and all methods forms the basis for the final written research manuscript. Guidelines already exist for preparing some types of research protocol, each linked with corresponding guidelines for reporting study findings. For example, the SPIRIT checklist (Chan *et al*, 2013) is used to guide the preparation of a protocol for a randomised controlled trial, with much overlap of concepts and structure with the CONSORT checklist (Schulz *et al*, 2010) for reporting trial findings.

## Oncology-specific reporting guidelines

The EQUATOR Network website already offers important resources to help authors write up oncology research studies. In October 2016 our regularly updated database of reporting guidelines included 37 oncology-specific guidelines. These guidelines complement the generic guidelines, offering guidance on reporting aspects of various study types, such as observational studies, prognostic and diagnostic studies, and clinical trials.

Table 3 describes the 37 oncology-specific guidelines by the clinical area, study type, and section of the report that they refer to. Some cover oncology studies in general, while others focus on research in certain diagnostic groups, such as cancer of the lung, liver, breast, kidney, bone, and soft tissue. The fields of haematology, neuro-oncology, urology, and gastroenterology are well-represented.

## Use of oncology-specific reporting guidelines

The publication of a reporting guideline will not affect reporting completeness and quality unless researchers working in the field are aware of and use the guideline when they write their manuscripts. We investigated how many times each oncology-specific reporting guideline had been cited by other research papers. We searched the Web of Science Core Collection Science Citation Index Expanded (SCI-EXPANDED) from inception to the present (last search date 3 November 2016). The results of these citation searches are shown in Table 3.

Two guidelines have each been cited more than 1000 times. Both focus on haematology research, one covering the whole study report for trials in a particular disease (acute myeloid leukaemia, (Cheson *et al*, 2003)) and the other dealing with a particular kind of data (correlating genetic and clinical data (Döhner *et al*, 2010)). Five have been cited between 100 and 350 times, and 23 have been cited less than 100 times. One guideline had no citations but was only published in 2016, and six guidelines were not found in the Citation Index.

It is likely that many authors who use a reporting guideline do not actually cite it, and that not all research papers that cite a guideline do so because it was used to help write the paper. Nevertheless, the citation numbers give a rough indication of the use of each guideline in the literature. Many factors will influence the differences between the citation rates of specific guidelines, for example the size of the subspecialty within oncology and when the guideline was published.

Journals have an important role to play in improving reporting of research studies by highlighting the use of reporting guidelines in their instructions to authors. Reporting guidelines can also be very helpful for peer-reviewers. The EQUATOR Network has produced a new toolkit to help journals to publish clear and therefore usable research reports (http://www.equator-network.org/toolkits/using-guidelines-in-journals/).

## Future work of EQUATOR Oncology

Health research reporting problems have been well-documented in recent years, and oncology research is no exception (Papathanasiou and Zintzaras, 2010; Peron *et al*, 2012, 2013; Jankova *et al*, 2015; Maillet *et al*, 2016; Sivendran and Galsky, 2016). Despite these continuing reporting issues, two specific guidelines for haematology research have been very well cited. The question remains as to why authors do not use existing reporting guidelines. Is it because authors need additional specific guidelines for certain oncology study types, because authors need help to better use existing guidelines (both generic and specific), because they do not think reporting guidelines are worth the effort, or because they are simply unaware that reporting guidelines exist?

EQUATOR Oncology aims to highlight the real-life consequences of poor reporting, to provide resources and to support oncology researchers by helping them to find and use the appropriate reporting guidelines for their research.

Peer-reviewers also play an important role in the process of improving research reporting, and reporting guidelines can be a helpful tool for those reviewing manuscripts prior to publication. However, checking adherence to a reporting guideline can be time-consuming. It may be helpful for guideline developers to produce short lists of items for peer reviewers to focus their attention on.

We will establish an EQUATOR Oncology ‘Advisory Group’ of experts and opinion leaders in oncology research including clinicians, oncologists, methodologists, editors, Cancer Research UK and EQUATOR representatives – who will oversee and inform the development of the project. Based on our findings from literature reviews and advice from our expert advisory group, we will identify issues and develop oncology-specific online resources to help minimise reporting problems and increase the impact of published oncology research.

We will also carry out surveys of oncology researchers, oncology journal editors and peer reviewers to identify their concerns about reporting in journal articles.

Our web resources can help oncology researchers improve their research reporting in manuscripts and will ultimately help to improve the robustness and reliability of the research itself. Only with the expert help of authors, researchers, methodologists, opinion leaders, and journal editors working in cancer research can our online resources fully address the reporting issues that oncology researchers need assistance with. We encourage those involved in cancer research to contact us with suggestions for the development of these resources.

Cancer patients take part in research studies to try to improve their health conditions and for altruistic reasons (Moorcraft *et al*, 2016). Their contributions to scientific understanding should not be wasted because the research is poorly conducted, inadequately reported, or even not reported at all. EQUATOR Oncology will support oncology researchers to conduct robust research and to produce research papers that are usable, reproducible, and transparent, recognising the important contributions of all patient participants in research.

## Figures and Tables

**Table 1:** Examples of reporting problems in oncology studies

**Reporting issue**	**Examples of poor reporting**	**Consequences of poor reporting**	**Reference**
**Observational studies**			
Prognostic studies not published	Completed prognostic studies of biomarkers not published.	Conclusions drawn from systematic reviews may be inaccurate due to the evidence base not being complete (likely publication bias).	Sekula *et al* (2016)
Epidemiological studies: incomplete reporting of methodology	Under-reporting of methodological aspects of observational studies including: matching, absolute risks, lack of flow diagram and missing data.	Difficult for readers to assess the validity of the studies.	Papathanasiou and Zintzaras (2010)
Prognostic factor studies: methods poorly reported	Inadequate reporting of aspects of study design and implementation in studies of prognostic markers, including: power calculations, time of enrolment, lists of candidate variables, definition of outcomes and providing the assay reference.	Studies are often too small to detect modest effects, and results from a number of studies may be examined together in systematic reviews or meta-analyses. If methods and findings are not reported in sufficient detail, it is not possible to include studies in such reviews.	Kyzas *et al* (2007)
**Clinical trials: results**			
Results not published	Trial findings that have been presented at professional meetings remain unreported or there is a delay in reporting.	Publication bias limits the available evidence base and, if decision to not publish is driven by results, distorts the overall evidence picture (likely publication bias). This can lead to treatments being used based on overoptimistic published results.	Tam *et al* (2011)
Inconsistencies within publications	Differences between reporting in abstracts and the main body of the text of the published articles: for example, strong support for the experimental arm of the study in the abstract, but not in the main text of the report.	Busy clinicians and policymakers may only read the abstract of the article. Reading only the published abstract may lead to a distorted view of the overall study findings, with implications for physicians when making decisions about clinical care.	Altwairgi *et al* (2012)
Data published but with deficiencies	Poor reporting of adverse event collection, description of AE characteristics leading to withdrawals, and whether AEs were attributed to trial interventions.	If information about effects of complex, (often combined) therapies are unavailable, could invalidate the decision-making process for clinicians and their patients.	Peron *et al* (2013)
	Poor reporting of adverse events in surgery, lack of standardised description of adverse events.	Physicians cannot assess the benefits and risks to patients that are likely to be offered surgery.	Meghelli *et al* (2016)
Poor reporting of trial outcomes	Trial outcomes: Selective trial outcome reporting, such as a discrepancy between the planned and published primary trial endpoints. Lack of reporting of planned non-primary trial endpoints.	Difficult to reproduce studies with poor reporting of outcomes. Overestimation of intervention effect sizes, which has an impact on evidence-based clinical decision making.	Raghav *et al* (2015)
	Patient-reported outcomes (PROs), quality of life: Poor reporting of methods of PRO collection and analysis, the pre-specified PRO hypothesis, methods for PRO collection and statistical approaches for dealing with missing data.	Patient-reported outcomes are essential in oncology trials. In conjunction with primary outcomes, such as survival, to allow the assessment of benefits and harms associated with the treatment. They are the ‘voice’ of the patients in the trial and therefore provide a unique perspective on the treatment; they should be addressed in the trial report.	Bylicki *et al* (2015)
**Clinical trials: methods**			
	Trial interventions **–** Chemotherapy: Poor reporting of the relative dose intensity, dose modification, early treatment discontinuation.	Replication and translation into clinical practice is impossible if there is not detailed information on the treatment administered under trial conditions.	Altwairgi *et al* (2015)

**Table 2:** Generic reporting guidelines available in the EQUATOR Network database of reporting guidelines

**Guideline abbreviation**	**Scope of reporting guideline**	**Link to further details about the guideline in the EQUATOR Network database**
CONSORT	Parallel group randomised trials (extensions address other designs)	Schulz *et al*, 2010, http://www.equator-network.org/reporting-guidelines/consort/
STROBE	Observational studies in epidemiology: cohort, case-control studies, cross-sectional studies	von Elm *et al*, 2007, http://www.equator-network.org/reporting-guidelines/strobe/
PRISMA	Systematic reviews and meta-analyses (in particular of randomised trials)	Moher *et al*, 2009, http://www.equator-network.org/reporting-guidelines/prisma/
CARE	Clinical case reports	Gagnier *et al*, 2014, http://www.equator-network.org/reporting-guidelines/care/
SRQR	Qualitative research	O'Brien *et al*, 2014, http://www.equator-network.org/reporting-guidelines/srqr/
COREQ	Qualitative research interviews and focus groups	Tong *et al*, 2007, http://www.equator-network.org/reporting-guidelines/coreq/
STARD	Diagnostic test accuracy studies	Bossuyt *et al*, 2015, http://www.equator-network.org/reporting-guidelines/stard/
TRIPOD	Studies developing, validating, or updating a prediction model, for either diagnosis or prognosis	Collins *et al*, 2015, http://www.equator-network.org/reporting-guidelines/tripod-statement/
REMARK	Tumour marker prognostic studies	McShane *et al*, 2005, http://www.equator-network.org/reporting-guidelines/reporting-recommendations-for-tumour-marker-prognostic-studies-remark/
SQUIRE	Quality improvement in health care	Ogrinc *et al*, 2016, http://www.equator-network.org/reporting-guidelines/squire/
CHEERS	Economic evaluations of health interventions	Husereau *et al*, 2013, http://www.equator-network.org/reporting-guidelines/cheers/
ARRIVE	Bioscience research using laboratory animals	Kilkenny *et al*, 2012, http://www.equator-network.org/reporting-guidelines/improving-bioscience-research-reporting-the-arrive-guidelines-for-reporting-animal-research/
SPIRIT	Clinical trial protocols	Chan *et al*, 2013, http://www.equator-network.org/reporting-guidelines/spirit-2013-statement-defining-standard-protocol-items-for-clinical-trials/
PRISMA-P	Systematic reviews and meta-analysis protocols	Moher *et al*, 2015, http://www.equator-network.org/reporting-guidelines/prisma-protocols/

**Table 3:** Oncology-specific reporting guidelines available on the EQUATOR website

**Guideline provided for**	**Clinical area of study that guideline relates to**	**Section of study report that guideline relates to**	**No of citations of guideline**	**Guideline available via 'Open Access'**	**Guideline reference and link to more information about the guideline in the EQUATOR database [number]**
**(a) Guidance for reporting all parts of a study**					
*1. Clinical trials*					
Myeloma clinical trials	Oncology, haematology	Whole report	186	Yes	Rajkumar *et al*, 2011 [1]
Phase 1 and phase 2 clinical trials in neuro-oncology	Oncology, neurology	Whole report	16	Yes	Chang *et al*, 2005 [2]
Surgically-based therapeutic clinical trials	Oncology, neurology, surgery	Whole report	8		Chang *et al*, 2007 [3]
Therapeutic trials in acute myeloid leukaemia (AML)	Oncology, haematology	Whole report	1149		Cheson *et al*, 2003 [4]
Clinical trials in cancer pain educational interventions	Oncology	Whole report	7	Yes	Stiles *et al*, 2010 [5]
*2. Observational studies*					
Tumour marker prognostic studies	Oncology, genetics	Whole report	313	Yes	McShane *et al*, 2005 [6]
The design and analysis of prognostic factor studies in non-small cell lung cancer (NSCLC)	Oncology, genetics, respiratory medicine	Whole report	151	Yes	Subramanian and Simon, 2010 [7]
**(b) Guidance for reporting certain parts of a study**					
*1. Clinical trials*					
Reporting of BCR-ABL molecular testing	Oncology, haematology	Procedure/method, results	4		Akard and Wang, 2011 [8]
Reporting clinical trial results on electrochemotherapy	Oncology	Procedure/method, study characteristics, intervention, results, data, outcomes, ethical issues, research recommendations	4	Yes	Campana *et al*, 2016 [9]
Standard definitions and endpoints for neoadjuvant clinical trials in breast cancer	Oncology	Terminology/definitions	23		Fumagalli *et al*, 2012 [10]
Clinical trials in systemic light-chain amyloidosis	Oncology, haematology	Procedure/method, study characteristics, outcomes	48		Comenzo *et al*, 2012 [11]
Clinical trials for patients in the state of a rising prostate-specific antigen	Oncology, urology	Study characteristics, outcomes	121	Yes	Scher *et al*, 2004 [12]
Flow cytometry minimal residual disease analysis and reporting in multiple myeloma	Oncology, haematology	Procedure/method, results	3	Yes	Arroz *et al*, 2016 [13]
Reporting system for correlation of cytogenetic and molecular genetic data with clinical data	Oncology, haematology	Procedure/method, intervention, outcomes	1094	Yes	Döhner *et al*, 2010 [14]
Reporting definitions, methodological and statistical issues for phase 3 clinical trials in chronic myeloid leukaemia	Oncology	Terminology/definitions, statistical methods and analyses	30	Yes	Guilhot *et al*, 2012 [15]
Clinical platelet transfusion studies	Oncology, haematology	Procedure/method, intervention, outcomes, harms/adverse effects/safety data	2		Meyer *et al*, 2013 [16]
Reporting embolisation treatment of vascular head, neck and brain tumours	Oncology, neurology, radiology, surgery	Terminology/definitions	10	Yes	Duffis *et al*, 2012 [17]
Reporting MRI evaluation of response after neoadjuvant radiotherapy in soft tissue sarcoma	Oncology, radiology	Images	0	Yes	Messiou *et al*, 2016 [18]
To promote standardisation and diminish variations in the acquisition, interpretation, and reporting of whole-body MRI scans for use in advanced prostate cancer	Oncology, urology, radiology, nuclear medicine	Images	Reference not found	Yes	Padhani *et al*, 2017 [19]
Reporting of post-neoadjuvant systemic therapy breast cancer specimens	Oncology, pathology	Procedure/method, study characteristics, terminology/definitions, intervention	4	Yes	Provenzano *et al*, 2015 [20]
Reporting of health-related quality of life in clinical cancer trials	Oncology	Outcomes	51		Lee and Chi, 2000 [21]
Use of historical data for determining "go/no go" decision for definitive phase III testing	Oncology	Procedure/method, data	32	Yes	Vickers *et al*, 2007 [22]
Common terminology criteria for paediatric reporting of adverse events in oncology trials	Oncology, paediatrics	Terminology/definitions, harms/adverse effects/safety data	Reference not found		Reeve *et al*, 2017 [23]
*2. Observational studies*					
Presenting prognostic studies with missing covariate data	Oncology	Data	87	Yes	Burton and Altman, 2004 [24]
Reporting case series of tumours of the colon and rectum	Oncology, gastroenterology, surgery	Procedure/method, study characteristics, intervention, results, data, outcomes	Journal indexed from 2008 onwards		Rubino and Pragnell, 1999 [25]
Reporting of individual MRI studies in men with prostate cancer on active surveillance and for reporting the outcomes of cohorts of men with prostate cancer having MRI on active surveillance	Oncology, urology, radiology	Images	Reference not found		Moore *et al*, 2017 [26]
*3. Other study types*					
Standards for balanced reporting on websites and in newspapers	Oncology, obstetrics & gynaecology		12		Bodemer *et al*, 2012 [27]
Reporting clinical studies of radioembolisation of hepatic malignancies	Oncology, gastroenterology, radiology	Procedure/method, study characteristics, terminology/definitions, intervention, images, outcomes, harms/adverse effects/safety data	78	Yes	Salem *et al*, 2011 [28]
Transcatheter therapies for hepatic malignancy	Oncology, gastroenterology, radiology, surgery	Procedure/method, study characteristics, terminology/definitions, outcomes, harms/adverse effects/safety data	51	Yes	Brown *et al*, 2009 [29]
Reporting clinical studies and research on the use of ablation methods for the treatment of benign bone tumours and metastases involving bone and soft tissues beyond the liver and lung	Oncology, radiology	Procedure/method, study characteristics, intervention, results, images, outcomes, harms/adverse effects/safety data	14	Yes	Callstrom *et al*, 2009 [30]
Percutaneous thermal ablation of primary renal cell carcinoma	Oncology, renal medicine, radiology, surgery	Procedure/method, study characteristics, terminology/definitions, intervention, images, outcomes, harms/adverse effects/safety data	19	Yes	Clark *et al*, 2009 [31]
Percutaneous vertebral augmentation	Oncology, rheumatology, radiology, surgery	Procedure/method, study characteristics, intervention, outcomes, harms/adverse effects/safety data	8	Yes	Radvany *et al*, 2009 [32]
Reporting the various aspects of image-guided ablation therapy	Oncology, radiology	Study characteristics, terminology/definitions, intervention, results, images, statistical methods and analyses, outcomes, harms/adverse effects/safety data	101	Yes	Ahmed *et al*, 2014 [33]
Reporting image-guided irreversible electroporation ablation therapy	Oncology, radiology	Terminology/definitions, data, images	Reference not found	Yes	Wendler *et al*, 2016 [34]
Reporting and gathering data on dose-volume dependencies of treatment outcome	Oncology	Data	65	Yes	Jackson *et al*, 2010 [35]
Calibration methods in cancer simulation models	Oncology	Procedure/method, data	38		Stout *et al*, 2009 [36]
Reporting HIF-1α-TG interactions	Oncology, genetics	Data	Reference not found		Slemc and Kunej, 2016 [37]

[1]http://www.equator-network.org/reporting-guidelines/consensus-recommendations-for-the-uniform-reporting-of-clinical-trials-report-of-the-international-myeloma-workshop-consensus-panel-1/

[2]http://www.equator-network.org/reporting-guidelines/gnosis-guidelines-for-neuro-oncology-standards-for-investigational-studies-reporting-of-phase-1-and-phase-2-clinical-trials/

[3]http://www.equator-network.org/reporting-guidelines/gnosis-guidelines-for-neuro-oncology-standards-for-investigational-studies-reporting-of-surgically-based-therapeutic-clinical-trials/

[4]http://www.equator-network.org/reporting-guidelines/revised-recommendations-of-the-international-working-group-for-diagnosis-standardization-of-response-criteria-treatment-outcomes-and-reporting-standards-for-therapeutic-trials-in-acute-myeloid-leuk/

[5]http://www.equator-network.org/reporting-guidelines/clinical-trials-focusing-on-cancer-pain-educational-interventions-core-components-to-include-during-planning-and-reporting/

[6]http://www.equator-network.org/reporting-guidelines/reporting-recommendations-for-tumour-marker-prognostic-studies-remark/

[7]http://www.equator-network.org/reporting-guidelines/gene-expression-based-prognostic-signatures-in-lung-cancer-ready-for-clinical-use/

[8]http://www.equator-network.org/reporting-guidelines/translating-trial-based-molecular-monitoring-into-clinical-practice-importance-of-international-standards-and-practical-considerations-for-community-practitioners/

[9]http://www.equator-network.org/reporting-guidelines/recommendations-for-improving-the-quality-of-reporting-clinical-electrochemotherapy-studies-based-on-qualitative-systematic-review/

[10]http://www.equator-network.org/reporting-guidelines/a-common-language-in-neoadjuvant-breast-cancer-clinical-trials-proposals-for-standard-definitions-and-endpoints/

[11]http://www.equator-network.org/reporting-guidelines/consensus-guidelines-for-the-conduct-and-reporting-of-clinical-trials-in-systemic-light-chain-amyloidosis/

[12]http://www.equator-network.org/reporting-guidelines/eligibility-and-outcomes-reporting-guidelines-for-clinical-trials-for-patients-in-the-state-of-a-rising-prostate-specific-antigen-recommendations-from-the-prostate-specific-antigen-working-group/

[13]http://www.equator-network.org/reporting-guidelines/consensus-guidelines-on-plasma-cell-myeloma-minimal-residual-disease-analysis-and-reporting/

[14]http://www.equator-network.org/reporting-guidelines/diagnosis-and-management-of-acute-myeloid-leukemia-in-adults-recommendations-from-an-international-expert-panel-on-behalf-of-the-european-leukemianet/

[15]http://www.equator-network.org/reporting-guidelines/definitions-methodological-and-statistical-issues-for-phase-3-clinical-trials-in-chronic-myeloid-leukemia-a-proposal-by-the-european-leukemianet/

[16]http://www.equator-network.org/reporting-guidelines/a-reporting-guideline-for-clinical-platelet-transfusion-studies-from-the-best-collaborative/

[17]http://www.equator-network.org/reporting-guidelines/head-neck-and-brain-tumor-embolization-guidelines/

[18]http://www.equator-network.org/reporting-guidelines/evaluation-of-response-after-pre-operative-radiotherapy-in-soft-tissue-sarcomas-the-european-organisation-for-research-and-treatment-of-cancer-soft-tissue-and-bone-sarcoma-group-eortc-stbsg-and-ima/

[19]http://www.equator-network.org/reporting-guidelines/metastasis-reporting-and-data-system-for-prostate-cancer-practical-guidelines-for-acquisition-interpretation-and-reporting-of-whole-body-magnetic-resonance-imaging-based-evaluations-of-multiorgan-i/

[20]http://www.equator-network.org/reporting-guidelines/standardization-of-pathologic-evaluation-and-reporting-of-postneoadjuvant-specimens-in-clinical-trials-of-breast-cancer-recommendations-from-an-international-working-group/

[21]http://www.equator-network.org/reporting-guidelines/the-standard-of-reporting-of-health-related-quality-of-life-in-clinical-cancer-trials/

[22]http://www.equator-network.org/reporting-guidelines/setting-the-bar-in-phase-ii-trials-the-use-of-historical-data-for-determining-gono-go-decision-for-definitive-phase-iii-testing/

[23]http://www.equator-network.org/reporting-guidelines/eliciting-the-childs-voice-in-adverse-event-reporting-in-oncology-trials-cognitive-interview-findings-from-the-pediatric-patient-reported-outcomes-version-of-the-common-terminology-criteria-for-adv/

[24]http://www.equator-network.org/reporting-guidelines/missing-covariate-data-within-cancer-prognostic-studies-a-review-of-current-reporting-and-proposed-guidelines/

[25]http://www.equator-network.org/reporting-guidelines/guidelines-for-reporting-case-series-of-tumours-of-the-colon-and-rectum/

[26]http://www.equator-network.org/reporting-guidelines/reporting-magnetic-resonance-imaging-in-men-on-active-surveillance-for-prostate-cancer-the-precise-recommendations-a-report-of-a-european-school-of-oncology-task-force/

[27]http://www.equator-network.org/reporting-guidelines/do-the-media-provide-transparent-health-information-a-cross-cultural-comparison-of-public-information-about-the-hpv-vaccine/

[28]http://www.equator-network.org/reporting-guidelines/research-reporting-standards-for-radioembolization-of-hepatic-malignancies/

[29]http://www.equator-network.org/reporting-guidelines/transcatheter-therapy-for-hepatic-malignancy-standardization-of-terminology-and-reporting-criteria/

[30]http://www.equator-network.org/reporting-guidelines/research-reporting-standards-for-image-guided-ablation-of-bone-and-soft-tissue-tumors/

[31]http://www.equator-network.org/reporting-guidelines/reporting-standards-for-percutaneous-thermal-ablation-of-renal-cell-carcinoma/

[32]http://www.equator-network.org/reporting-guidelines/research-reporting-standards-for-percutaneous-vertebral-augmentation/

[33]http://www.equator-network.org/reporting-guidelines/image-guided-tumor-ablation-standardization-of-terminology-and-reporting-criteria-a-10-year-update/

[34]http://www.equator-network.org/reporting-guidelines/irreversible-electroporation-ire-standardization-of-terminology-and-reporting-criteria-for-analysis-and-comparison/

[35]http://www.equator-network.org/reporting-guidelines/the-lessons-of-quantec-recommendations-for-reporting-and-gathering-data-on-dose-volume-dependencies-of-treatment-outcome/

[36]http://www.equator-network.org/reporting-guidelines/calibration-methods-used-in-cancer-simulation-models-and-suggested-reporting-guidelines/

[37]http://www.equator-network.org/reporting-guidelines/transcription-factor-hif1a-downstream-targets-associated-pathways-polymorphic-hypoxia-response-element-hre-sites-and-initiative-for-standardization-of-reporting-in-scientific-literature/

A version of Table 3 with active links is provided online as Supplementary Table 1.
